# Association between hearing protection device use and noise-induced hearing loss among manufacturing workers in China: a cross-sectional study

**DOI:** 10.3389/fpubh.2026.1787668

**Published:** 2026-04-13

**Authors:** Yuan Pan, Zhengheng Zhang, Wei Qiu, Meibian Zhang, Jinhao Wu, Xinxin Li, Youzhi Guan, Minhong Zhang, Yuying Wang, Weijiang Hu

**Affiliations:** 1 National Institute of Occupational Health and Poisoning Control, Chinese Center for Disease Control and Prevention, Beijing, China; 2 Research Center for Occupational Health, Yangtze Delta Region Institute of Tsinghua University, Jiaxing, Zhejiang, China; 3 Shenzhen Prevention and Treatment Center for Occupational Diseases, Shenzhen, Guangdong, China

**Keywords:** hearing protection device, high-frequency pure-tone average, noise-induced hearing loss, occupational noise, workers

## Abstract

**Objective:**

Noise-induced hearing loss (NIHL) is a leading occupational illness globally. This study aimed to identify factors related to hearing protection device (HPD) use that were associated with NIHL and to quantify the relationship between the duration of HPD use and NIHL risk.

**Methods:**

In a cross-sectional study, 845 noise-exposed manufacturing workers in China underwent health examinations and surveys. The associations between hearing protection devices use duration, related factors, and hearing loss were analyzed using multivariable linear regression and restricted cubic spline model.

**Results:**

Providing hearing protection and training in its use at the enterprise level were associated with less hearing loss. At the individual level, consistent full-day use of hearing protection and using earplugs instead of earmuffs were linked to significantly less hearing loss. Lower hearing thresholds were linked to longer HPD use: each additional year of use was associated with a modest (~0.3 dB) reduction in the increase of hearing thresholds. Greater protective associations were observed after approximately 3 years of continuous use.

**Conclusion:**

Longer duration of HPD use is associated with milder hearing loss, particularly among workers reporting sustained use for three or more years, underscoring the importance of long-term hearing protection in noisy workplaces.

## 1 Introduction

Noise is a pervasive occupational hazard globally. Noise-induced hearing loss (NIHL), a sensorineural condition caused by prolonged noise exposure, represents a significant irreversible occupational injury in industrial settings characterized by sustained high noise levels (1, 2). The condition impairs workers’ ability to perceive warning signals and disrupts communication (3), negatively impacting productivity and increasing the likelihood of occupational accidents (4). It also significantly affects an individual’s quality of life and the broader social economy (5). According to the National Institute for Occupational Safety and Health (NIOSH), approximately 25% of workers are exposed to hazardous noise, and 20% of those exposed show significant hearing impairment (6). In China, 88.8% of enterprises reported noise hazards in 2020, with an estimated 80 million workers in the industrial and service sectors exposed to hazardous noise (7). The Global Burden of Disease 2021 report emphasized the substantial burden of NIHL, with 7.8 million disability-adjusted life years (DALYs) globally, and China leading with 2.6 million NIHL-related DALYs (8). Unlike the inevitable age-related degeneration, NIHL is preventable through effective strategies and technologies (1). Effective prevention depends on identifying modifiable risk factors for NIHL and implementing targeted interventions.

Previous studies on NIHL risk factors can be categorized into occupational and non-occupational noise exposure. Historically, noise research has focused on equivalent continuous sound levels (Leq), based on the assumption in ISO 1999:2013 that hearing loss correlates with the total energy of the exposure (9). Current noise guidelines rely on the equal-energy hypothesis (EEH), which posits that all noise exposure with equivalent energy content carries the same risk, a concept considered valid for steady-state noise but inadequate for the temporal dynamics of complex noise exposure. Industrial workers are often subjected to such complex noise environments, which consist of both impulsive and continuous noise components (10). Consequently, recent studies have utilized kurtosis statistics to more accurately assess complex noise exposure (11, 12). Kurtosis provides insight into the complexity of noise and reflects the temporal structure of noise (10). Even when acoustic energies and spectra are similar, noises with different kurtosis values can result in vastly different hearing loss (13, 14), thereby influencing the accurate assessment of hearing protection device (HPD) effectiveness. Workers exposed to high-kurtosis noise experience more severe hearing loss, and HPD use may provide greater protective effects in such environments. Additionally, the duration of noise exposure and prolonged high-intensity noise exposure, referred to as cumulative noise exposure (CNE), are established risk factors for NIHL (15). Beyond occupational noise, various non-occupational factors contribute significantly to NIHL risk. These include genetic variations (1), sociodemographic characteristics such as age, gender, ethnicity, and income level (16), lifestyle factors including smoking, drinking (17), engagement in high-noise recreational activities (e.g., shooting, use of high-volume audio devices), and physical activity (18–20), exposure to ototoxic agents (21), and health conditions such as blood pressure, cholesterol levels (19), ear infections (16), and head injuries (22). Currently, no unified standard exists for assessing NIHL. Some studies have used thresholds at 3, 4, and 6 kHz due to their sensitivity to early noise-induced damage (23, 24), while others have used thresholds at 0.5, 1, 2, and 4 kHz to evaluate NIHL (25, 26). Although the frequency ranges vary across studies, a 25 dB cutoff has been widely adopted as the threshold for defining NIHL (23, 27). To protect workers at high risk of NIHL from frequent exposure to hazardous noise, many employers implement Hearing Conservation Programs (HCPs), which involve engineering controls, fitting HPDs, and other preventive strategies (2). When engineering controls fail to sufficiently lower noise levels at the source, HPDs remain the most common and essential method to reduce workers’ noise exposure (28).

The use of HPDs is a key factor in reducing NIHL risk. Zhang et al. (29) identified the frequency of HPD use as a significant predictor of NIHL in their predictive model. A recent systematic review demonstrated that enforced HPD use resulted in a significant reduction in NIHL (2). While the importance of HPDs in protecting against occupational noise exposure is widely recognized, discrepancies persist in evaluating their association with NIHL risk. Many studies have relied on simplified categorical assessments of HPD use (e.g., “Yes” or “No”; or, among users, classifying frequency as “Barely,” “Occasional,” “Basic,” or “Long-term”) (15, 18, 29), and these assessments may not fully capture the association between HPD use and NIHL, potentially resulting in miscalculations of observed relationships. Therefore, to better assess associations between HPD use and NIHL, quantitative metrics are necessary to surpass traditional qualitative classifications.

Given that few studies have focused on how NIHL changes with cumulative HPD use duration, this study provides a preliminary quantitative assessment of the association between cumulative HPD use duration and NIHL risk. Additionally, to further explore other HPD-related factors associated with NIHL, this study examined several qualitative HPD-related variables at both the enterprise and individual levels and their association with NIHL.

## 2 Materials and methods

### 2.1 Data sources and study population

This study utilized an analytical cross-sectional design, including 845 workers from the Workers Health Examination Database (WHED) (29) in Shenzhen, Guangdong Province, China. Individual exposure data for the workers were collected through field investigation and linked to the WHED using the unique enterprise credit code, the worker’s name, and identity card number.

The WHED is derived from an ongoing annual occupational disease surveillance program led by the National Institute of Occupational Health and Poison Control, Chinese Center for Disease Control and Prevention (CCDC). This program systematically gathers individual-level data from regional occupational disease prevention institutes across all 31 provinces in China. The collected data includes workers’ demographic information (e.g., name, identity card number, age, gender), occupational history (e.g., factory, industry type, unique credit code, worksite, employment duration, job type, and exposure to occupational hazards such as noise, organic solvents, heavy metals, and dust), and health examination results (e.g., pure-tone air-conduction audiometry, systolic blood pressure, diastolic blood pressure, height, and weight). The primary objective of this program is to assess the occupational disease status of Chinese workers and provide evidence-based support for public health policy development.

Based on previous monitoring reports, we first selected 26 typical manufacturing factories in Shenzhen, Guangdong Province. Workers in these factories were employed in six typical manufacturing industries, including machinery manufacturing, smelting and rolling, pharmaceutical and chemical, metal structure manufacturing, non-metallic mineral products manufacturing, and electrical, communication and instrument manufacturing. After communicating with the factory managers, two factories refused to participate, leaving 24 factories that agreed to participate. Subsequently, we requested and obtained data from the WHED database for all workers in these 24 factories, with a total of 1,999 workers.

Workers were initially included applying a consecutive sampling approach from the WHED database, based on following criteria: occupational exposure to noise [L_Aeq,8h_ ≥ 70 dB(A)] without concurrent exposure to organic solvents or heavy metals; age between 20 and 65 years. Following these, 1,689 eligible workers were invited for a cross-sectional field investigation.

### 2.2 Field investigation

Face-to-face questionnaires were administered by occupational hygienists to gather comprehensive information on workers’ demographics (e.g., name, identity card number, age, gender, monthly income), health behaviors (e.g., smoking, alcohol consumption, physical activity), health status (e.g., hereditary conditions, ear disease history, use of ototoxic medications, hypertension, diabetes), and occupational history (e.g., factory, employment duration, job type, daily noise exposure duration, and HPD-related factors) to construct detailed exposure profiles. HPD-related data were collected based on participants’ self-reported information regarding enterprise-level measures (e.g., provision of HPDs, training in their proper use) and individual-level practices (e.g., HPD use, daily use frequency, HPD type, and duration of use).

Currently, obtaining noise energy levels and kurtosis values requires field measurements with professional instruments to collect raw noise recordings from workers, followed by analysis using established formulas. For this study, full-shift noise recordings were conducted for each noise-exposed worker in 24 factories using a personal noise dosimeter (ASV5910-R, Hangzhou Aihua Instruments Co., Ltd., Hangzhou, China). We collected noise recordings from workers starting at the beginning of the shift; due to practical constraints, the actual recording durations ranged from 4 to 8 h. Although these recordings may not fully capture all potential intra-shift variability, inquiries and observations indicated that the workflow, workers’ processes, locations, and operating procedures were relatively consistent throughout the shift, and daily occupational noise exposure to workers was generally repetitive (29, 30). On this basis, a continuous recording of approximately half a shift (about 4 h) was considered reasonable to represent each worker’s full-shift (8 h) noise exposure.

The ASV5910-R is a specialized acoustic recording device designed for precise personal noise exposure measurements and analysis. It features a 1/4-inch pre-polarized condenser microphone with a wide frequency response of 20 Hz–20 kHz and a high sensitivity of 2.24 mV/Pa. The device offers a measurement range of 40–141 dB(A) and supports continuous operation for up to 23 h on a full charge. During measurements, the microphone was securely attached to the participant’s shoulder at the beginning of the work shift using a dedicated clip and retrieved at the shift’s end. Noise was continuously recorded at a sampling rate of 48 kHz with a 32-bit resolution. Investigators supervised the noise collection process in the workplace, with the recordings stored on 32-GB microSD memory cards. The questionnaires and audio recordings were collected between January and June 2025. The collected data were then transferred to a computer for storage and analysis.

The A-weighted 8-h equivalent continuous sound pressure level (L_Aeq,8h_) for each worker’s recording period was calculated as an indicator of noise energy, while the geometric mean kurtosis of consecutive non-overlapping 60-s windows was computed to characterize the temporal structure of noise and used as the final kurtosis metric. Both calculations were performed using Python (a statistical programming language, version 3.10.18). The audio recordings were imported into Python to extract the time-specific sound energy intensity (i.e., Leq). Kurtosis metrics were subsequently computed based on these Leq values. Detailed computational procedures are outlined in Supplementary Method 1.

During the field work, 634 of the 1,689 invited workers did not attend, and data were collected from 1,055 workers. Among them, 71 workers experienced data linkage failure, leaving 984 workers with successfully matched occupational surveillance audiometry, questionnaire-based information, and noise recordings.

**Figure 1:**
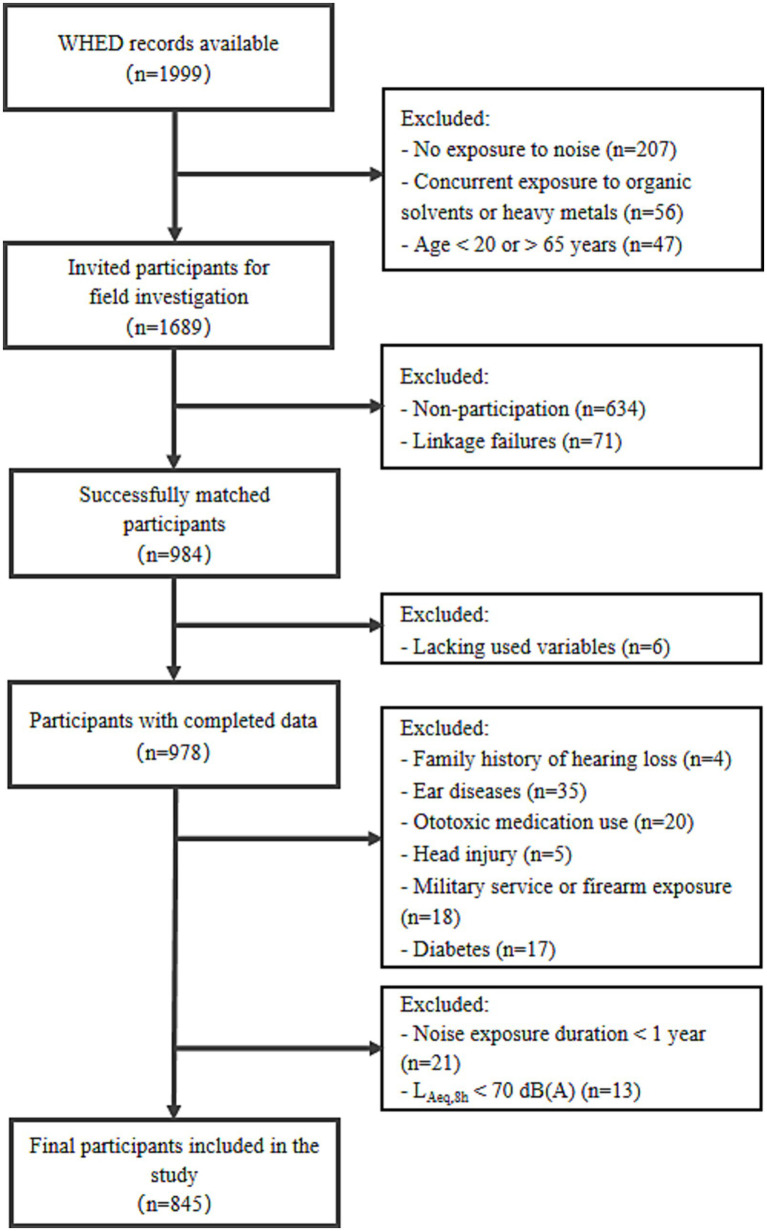
Flow chart of the study.

Participants were further selected based on the following inclusion and exclusion criteria. Inclusion criteria: (1) at least 1 year of employment at the current workplace; (2) L_Aeq,8h_ ≥ 70 dB(A). Exclusion criteria: (1) family history of hearing loss; (2) history of ear disease, use of ototoxic medications, or head injury; (3) history of military service or firearm use; (4) self-reported diabetes; (5) missing data on any study variables required for analysis. Workers exposed to L_Aeq,8h_ ≥ 70 dB(A) were selected because previous research (31) indicated that the recommended 8-h exposure limit of 85 dB(A) (32) may be insufficient, particularly for complex noise with impulsive components, highlighting the need to examine the biological effects of lower exposure levels. Furthermore, as Zhang et al. (14) noted in the limitation, additional data in the 70–78 dB(A) range are required. After applying these criteria, 845 participants out of an initial 984 were included in the final analysis. The selection process is shown in Figure 1. These participants were distributed across 24 factories in Shenzhen. Descriptive statistics stratified by factory are provided in Supplementary Table 1.

The study protocol was reviewed and approved by the Ethics Review Committee of the National Institute of Occupational Health and Poison Control, CCDC on March 7, 2025 (Approval No. NIOHP202518). Written informed consent was obtained from all participants after they were informed of the study’s objectives and design.

### 2.3 Audiometric evaluation and definition of outcome

The hearing loss metrics for this study were based on air-conduction pure-tone audiometry results obtained from the WHED, collected between January and December 2024. Each worker underwent a routine otologic examination, starting with otoscopy to confirm the absence of external ear abnormalities. Air-conduction pure-tone hearing thresholds were then measured at 0.5, 1, 2, 3, 4, and 6 kHz in both ears by an experienced audiologist. The audiometry was conducted in accordance with GB/T 7583 and ISO 8253-1 standards. In line with the diagnostic criterion (GBZ 49–2014) in China, each worker underwent at least three pure-tone audiometric assessments, with a minimum interval of 3 days between tests. Threshold deviations across frequencies were required to be less than 10 dB. For diagnostic grading, the lowest threshold from the three measurements at each frequency was used. The pure-tone threshold data derived from the WHED database had already been corrected for age and sex in accordance with the Chinese national standard (GB/T 7582). Audiograms were taken at least 16 h after the workers’ last occupational noise exposure.

The primary outcome for this study was defined as the average binaural high-frequency pure-tone average (HF-PTA) at 3, 4, and 6 kHz, which are the frequencies most sensitive for NIHL assessment (11, 23, 33), calculated using the following formula:

$$\mathrm{HF}-\mathrm{PTA}=\frac{HL_{L}+HL_{R}}{6}$$

where *HL*_L_ and *HL*_R_ represent the sum of hearing threshold levels (in dB) at 3,000, 4,000, and 6,000 Hz for the left and right ear, respectively.

### 2.4 Covariates

Sociodemographic characteristics (age, gender, monthly personal income), lifestyle and health status (smoking status, smoking duration, drinking status, drinking duration, physical activity, earphone use, body mass index [BMI], hypertension), and occupational noise exposure metrics (employment duration, L_Aeq,8h_, geometric kurtosis) were collected and included as covariates in the analysis. The selection of these covariates was guided by prior research identifying potential confounding factors related to NIHL (10, 18, 34–36).

Monthly personal income was categorized as <5,000 CNY, 5000–7,000 CNY, and >7,000 CNY. Smoking status was categorized into two groups (yes/no), with “yes” indicating that participants had smoked at least one cigarette per day for more than 6 months, including both current and former smokers. Smoking duration referred to the total number of years of smoking for both current and former smokers. Similarly, drinking status was categorized into two groups (yes/no), with “yes” indicating that participants had consumed alcoholic beverages at any point. Drinking duration referred to the total years of alcohol consumption for both current and former drinkers. Regular physical activity was defined as participation in exercise more than three times per week, with each session lasting at least 30 min. Earphone users were defined as individuals who had regularly used earphones for entertainment for at least 1 h per day during the past 6 months. BMI was calculated as weight in kilograms divided by the square of height in meters. Overweight was defined as a BMI ≥ 24.0 kg/m^2^ according to the Expert Consensus on Obesity Prevention and Treatment in China (37). Hypertension was defined as clinic systolic blood pressure ≥ 140 mmHg or diastolic blood pressure ≥ 90 mmHg, or current use of antihypertensive medication, as per the China Hypertension Prevention and Treatment Guidelines Revision Committee (2024) (38). Diabetes status was self-reported in the present study. Workers were asked, “Do you have a history of diabetes?” (Yes/No). Self-reported diabetes is a commonly used approach for determining diabetes in epidemiological studies and shows high consistency with gold-standard diagnostic criteria (39, 40).

### 2.5 Statistical analysis

HF-PTA was initially categorized into two groups for descriptive comparisons: a lower group (≤25 dB) and a higher group (>25 dB). In occupational hearing research, high-frequency hearing loss (typically at 3, 4, and 6 kHz) is often the earliest manifestation of noise-induced damage. Previous studies have adopted the 25 dB threshold at these frequencies to define NIHL, creating a descriptive categorization sensitive to high-frequency NIHL (23, 27). Accordingly, we used the average threshold at 3, 4, and 6 kHz and dichotomized participants into “lower” (≤25 dB) and “higher” (>25 dB) groups. Continuous variables are presented as mean ± standard deviation (SD) and range. Categorical variables are presented as counts and percentages (*n*, %). The Student’s *t*-test and Mann–Whitney *U* test were used to assess differences in continuous variables between the two groups, while Pearson’s chi-squared test was applied to categorical variables.

To minimize information loss associated with categorizing continuous variables (29), the continuous HF-PTA value was used as the outcome variable in subsequent modeling analyses. Linear regression models were first constructed to examine the associations between HPD-related factors and HF-PTA, with regression coefficients (*β*) and 95% confidence intervals (CIs) reported. Multivariable linear regression analyses were then performed to control for potential confounders.

Restricted cubic spline (RCS) was then applied to explore the linear or nonlinear association between HPD use duration and HF-PTA, with three knots placed at the 10th, 50th (median), and 90th percentiles of the HPD use duration distribution. This approach, consistent with other studies, ensured adequate model flexibility and stability (41, 42). Two-stage linear regression models were constructed by analyzing data on both sides of the inflection point, with covariate adjustments aligned with the previously described models. Subgroup analyses and interaction tests were conducted to assess consistency across different subpopulations.

All statistical analyses were performed using R (version 4.4.1).

## 3 Results

### 3.1 Participants’ characteristics

Among the 845 participants, the mean age was 39.09 ± 7.85 years, with 74.20% male and 25.80% female. Compared to the lower HF-PTA group, individuals in the higher HF-PTA group were more likely to be older (37.74 ± 7.71 vs. 41.06 ± 7.63 years; *p* < 0.001), had a higher proportion of males (67.80% vs. 83.48%; *p* < 0.001), had higher smoking rates (33.80% vs. 41.16%; *p* < 0.05), and had longer durations of both smoking (3.51 ± 6.23 vs. 4.57 ± 7.23 years; *p* < 0.05) and drinking (2.53 ± 5.74 vs. 3.26 ± 6.30 years; *p* < 0.05). Occupational noise exposure profiles showed that participants in the higher HF-PTA group had longer exposure durations (6.82 ± 5.75 vs. 8.13 ± 5.77 years; *p* = 0.001) and higher L_Aeq,8h_ (81.7 ± 5.9 vs. 83.5 ± 5.7 dB(A); *p* < 0.001) than those in the lower group (Table 1).

**Table 1:** Descriptive characteristics of the study participants.

Characteristics	Overall	HF-PTA (dB HL)		*p*-value
		Lower (≤25)	Higher (>25)	
	*N* = 845	*N* = 500	*N* = 345	
Sociodemographic				
Age, years	20–61	20–58	21–61	<0.001
	39.09 ± 7.85	37.74 ± 7.71	41.06 ± 7.63	
Gender, *n* (%)				<0.001
Male	627 (74.20)	339 (67.80)	288 (83.48)	
Female	218 (25.80)	161 (32.20)	57 (16.52)	
Monthly income, CNY				0.318
<5,000	55 (6.51)	36 (7.20)	19 (5.51)	
5,000–7,000	539 (63.79)	324 (64.80)	215 (62.32)	
>7,000	251 (29.70)	140 (28.00)	111 (32.17)	
Lifestyle and health status				
Smoking, *n* (%)				0.035
Yes	311 (36.81)	169 (33.80)	142 (41.16)	
No	534 (63.20)	331 (66.20)	203 (58.84)	
Smoking duration, years	0–40	0–35	0–40	0.025
	3.94 ± 6.67	3.51 ± 6.23	4.57 ± 7.23	
Drinking, *n* (%)				0.081
Yes	243 (28.76)	132 (26.40)	111 (32.17)	
No	602 (71.24)	368 (73.60)	234 (67.83)	
Drinking duration, years	0–36	0–36	0–31	0.048
	2.83 ± 5.99	2.53 ± 5.74	3.26 ± 6.30	
Physical activity, *n* (%)				0.586
Yes	347 (41.07)	201 (40.20)	146 (42.32)	
No	498 (58.94)	299 (59.80)	199 (57.68)	
Earphone use, *n* (%)				0.675
Yes	243 (28.76)	147 (29.40)	96 (27.83)	
No	602 (71.24)	353 (70.60)	249 (72.17)	
BMI (kg/m^2^)	16.97–39.54	16.97–39.54	17.18–38.28	0.318
	23.71 ± 3.33	23.62 ± 3.35	23.84 ± 3.31	
Hypertension, *n* (%)				0.512
Yes	166 (19.65)	94 (18.80)	72 (20.87)	
No	679 (80.36)	406 (81.20)	273 (79.13)	
Occupational noise exposure				
Exposure duration, years	1–29	1–29	1–29	0.001
	7.36 ± 5.79	6.82 ± 5.75	8.13 ± 5.77	
L_Aeq,8h_[dB(A)]	70.0–105.4	70.0–102.6	70.1–105.4	<0.001
	82.4 ± 5.9	81.7 ± 5.9	83.5 ± 5.7	
Kurtosis	2.10–130.33	2.10–130.33	2.98–110.65	0.097
	10.80 ± 11.20	10.45 ± 11.06	11.31 ± 11.41	
HPD related				
Enterprise				
Provision, *n* (%)				0.413
Yes	808 (95.62)	481 (96.20)	327 (94.78)	
No	37 (4.38)	19 (3.80)	18 (5.22)	
Training, *n* (%)				0.454
Yes	761 (90.06)	454 (90.80)	307 (88.99)	
No	84 (9.94)	46 (9.20)	38 (11.01)	
Individual				
Use, *n* (%)				0.564
Yes	764 (90.41)	455 (91.00)	309 (89.57)	
No	81 (9.59)	45 (9.00)	36 (10.43)	
Daily use frequency, *n* (%)				0.093
No use	81 (9.59)	45 (9.00)	36 (10.44)	
Partially use	89 (10.53)	44 (8.80)	45 (13.04)	
Fully use	675 (79.88)	411 (82.2)	264 (76.52)	
Type, *n* (%)				0.748
No HPD	81 (9.59)	45 (9.00)	36 (10.44)	
Earplug	717 (84.85)	426 (85.20)	291 (84.35)	
Earmuff	47 (5.56)	29 (5.80)	18 (5.22)	
Use duration, years	0–29	0–29	0–29	0.166
	4.88 ± 4.76	4.72 ± 4.67	5.12 ± 4.89	

HF-PTA, high-frequency pure-tone average; HL, threshold level; HPD, hearing protection device; BMI, body mass index.

### 3.2 Association between HPD-related factors and HF-PTA

Three linear regression models were constructed to examine the association between HPD-related factors and HF-PTA (Table 2). Specifically, Enterprise-level HPD provision and training in their proper use were consistently associated with lower HF-PTA across all models, compared to the absence of such provision or training. At the individual practice level, after adjusting for all covariates in Model 3, participants reporting HPD use had significantly lower HF-PTA than non-users (*β* = −3.32, 95% CI: −5.99 to −0.64, *p* < 0.05). Furthermore, participants with daily full use of HPDs were associated with lower HF-PTA compared to those who did not use HPDs (*β* = −3.79, 95% CI: −6.48 to −1.10, *p* < 0.01). Similarly, participants using earplugs showed significantly lower HF-PTA than non-users (*β* = −3.40, 95% CI: −6.09 to −0.71, *p* < 0.05). However, no significant association with HF-PTA was observed for workers with partial HPD use or those using earmuffs compared to non-users.

**Table 2:** Relationship between the HPD-related factors and HF-PTA in different models.

Variables		Model 1		Model 2		Model 3	
		*β* (95% CI)	*p*-value	*β* (95% CI)	*P*-value	*β* (95% CI)	*P*-value
Enterprise							
Provision							
No		Reference		Reference		Reference	
Yes		−4.57 (−8.42, −0.73)	0.020*	−5.54 (−9.30, −1.78)	0.004*	−7.45 (−11.35, −3.55)	<0.001*
Training							
No		Reference		Reference		Reference	
Yes		−3.37 (−6.00, −0.74)	0.012*	−3.47 (−6.03, −0.90)	0.008*	−4.03 (−6.62, −1.44)	0.002*
Individual							
Use							
No		Reference		Reference		Reference	
Yes		−2.40 (−5.08, 0.28)	0.079	−2.13 (−4.74, 0.49)	0.111	−3.32 (−5.99, −0.64)	0.015*
Daily use frequency							
No use		Reference		Reference		Reference	
Partially use		1.02 (−2.49, 4.52)	0.569	0.20 (−3.24, 3.64)	0.908	−0.47 (−3.94, 3.01)	0.792
Fully Use		−2.85 (−5.53, −0.17)	0.037*	−2.46 (−5.09, 0.17)	0.067	−3.79 (−6.48, −1.10)	0.006*
Type							
No HPD		Reference		Reference		Reference	
Earplugs		−2.40 (−5.09, 0.28)	0.080	−2.13 (−4.76, 0.49)	0.111	−3.40 (−6.09, −0.71)	0.013*
Earmuffs		−2.32 (−6.53, 1.88)	0.278	−2.02 (−6.13, 2.09)	0.335	−2.40 (−6.51, 1.72)	0.253
Use duration, years							
Continuous		−0.01 (−0.17, 0.16)	0.920	−0.15 (−0.32, 0.02)	0.093	−0.27 (−0.48, −0.06)	0.011*
Quartiles							
0	(No use)	Reference		Reference		Reference	
≤1.5	(Q1)	−3.15 (−6.24, −0.07)	0.045*	−2.11 (−5.14, 0.92)	0.173	−2.94 (−6.05, 0.18)	0.065
1.5–3	(Q2)	−2.88 (−5.73, −0.03)	0.048*	−2.40 (−5.20, 0.40)	0.093	−3.22 (−6.13, −0.31)	0.030*
3–7	(Q3)	−2.74 (−5.54, 0.06)	0.055	−2.22 (−4.95, 0.52)	0.112	−3.13 (−5.95, −0.32)	0.029*
>7	(Q4)	−1.95 (−4.83, 0.93)	0.184	−2.71 (−5.58, 0.16)	0.064	−4.27 (−7.38, −1.16)	0.007*

Model 1: Unadjusted for any covariates.

Model 2: Adjusted for sociodemographic factors (age, gender, and monthly income).

Model 3: Adjusted for sociodemographic factors (age, gender, and monthly income), lifestyle habits and health status factors (smoking, smoking duration, drinking, drinking duration, physical activity, earphone use, BMI, and hypertension), and occupational noise exposure factors (exposure duration, L_Aeq,8h_, and kurtosis).

Q, quartile; CI, confidence interval.

*Statistically significant, with *p* < 0.05.

When HPD use duration was treated as a continuous variable, Model 3 showed that each additional year of HPD use was associated with a 0.27 dB decrease in HF-PTA (*β* = −0.27, 95% CI: −0.48 to −0.06, *p* < 0.05). In the quartile analysis, compared to the non-use reference group, participants in Quartiles 2 (1.5–3 years), 3 (3–7 years), and 4 (>7 years) showed lower HF-PTA (*β* = −3.22, 95% CI: −6.13 to −0.31, *p* < 0.05; *β* = −3.13, 95% CI: −5.95 to −0.32, *p* < 0.05; *β* = −4.27, 95% CI: −7.38 to −1.16, *p* < 0.01, respectively). However, no significant association with HF-PTA was observed in Quartile 1 (≤1.5 years of HPD use).

### 3.3 Nonlinear correlation analysis

**Figure 2:**
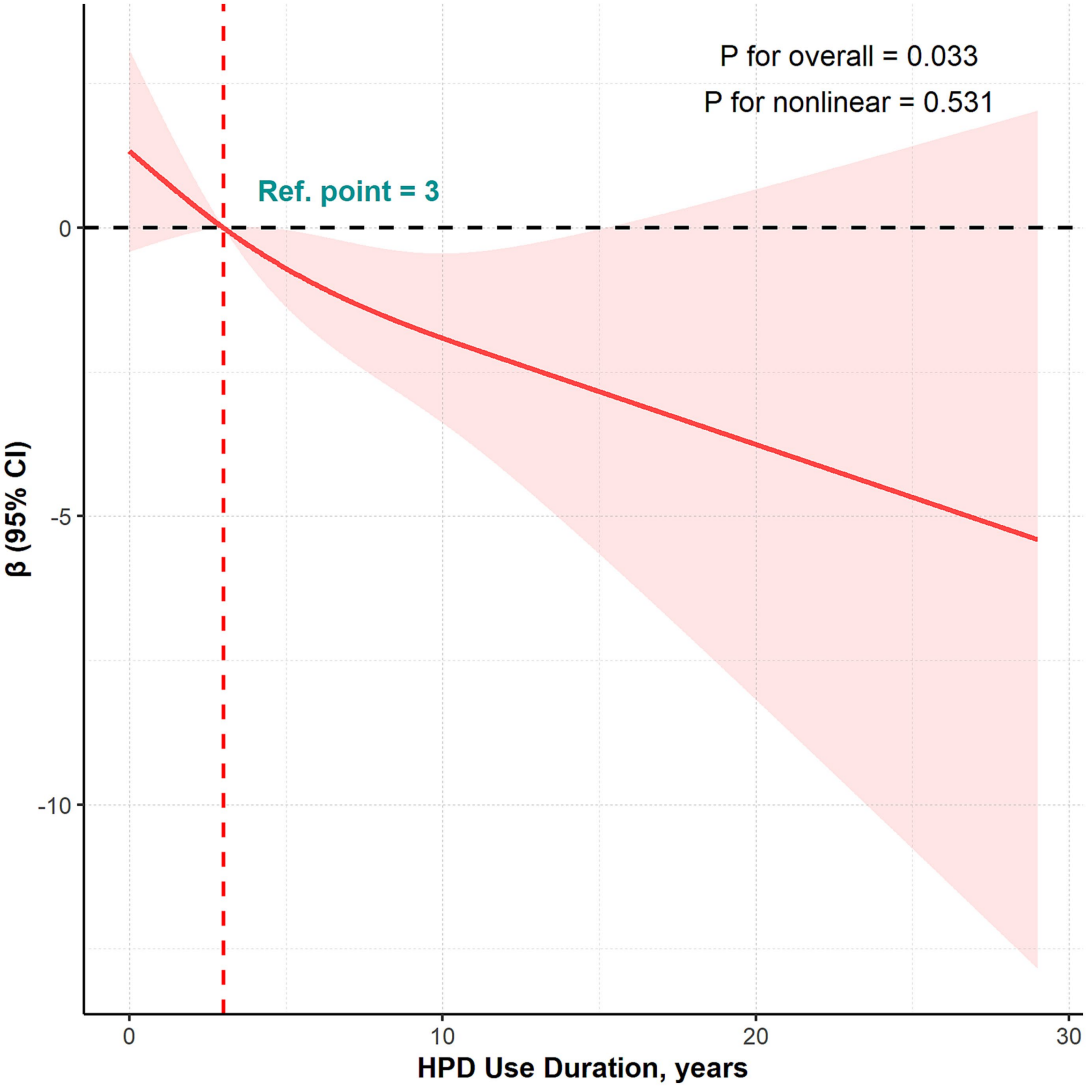
RCS curves illustrating the dose–response relationship between HPD use duration and HF-PTA after adjusting for covariates. The heavy central line represents the estimated adjusted *β*, with the shaded ribbons indicating the 95% CI. The model was adjusted for sociodemographic factors (age, gender, and monthly income), lifestyle and health status factors (smoking, smoking duration, drinking, drinking duration, physical activity, earphone use, BMI, and hypertension), and occupational noise exposure factors (exposure duration, L_Aeq,8h_, and kurtosis).

In the RCS analysis, HPD use duration demonstrated a significant linear inverse relationship with HF-PTA (*P* for non-linear > 0.05; Figure 2) in the overall population. The approximately three-year inflection point was identified in the analysis. The count distribution of HPD use duration for workers included in this study is provided in Supplementary Figure 1.

According to Model 3, for participants using HPDs for 3 years or longer, each additional year of HPD use was associated with a 0.32 dB reduction in HF-PTA (*β* = −0.32, 95% CI: −0.62 to −0.01, *p* < 0.05). However, no statistically significant association was found for participants using HPDs for less than 3 years (Table 3).

**Table 3:** Threshold effect analysis of the correlation between individual HPD use duration and HF-PTA.

Individual HPD use duration, years	Model 1		Model 2		Model 3	
	*β* (95% CI)	*P*-value	*β* (95% CI)	*P-*value	*β* (95% CI)	*P-*value
<3	−0.88 (−2.36, 0.60)	0.242	−0.71 (−2.13, 0.72)	0.329	−0.84 (−2.39, 0.70)	0.284
≥3	0.09 (−0.13, 0.31)	0.432	−0.08 (−0.33, 0.17)	0.523	−0.32 (−0.62, −0.01)	0.041

### 3.4 Subgroup analysis

**Figure 3:**
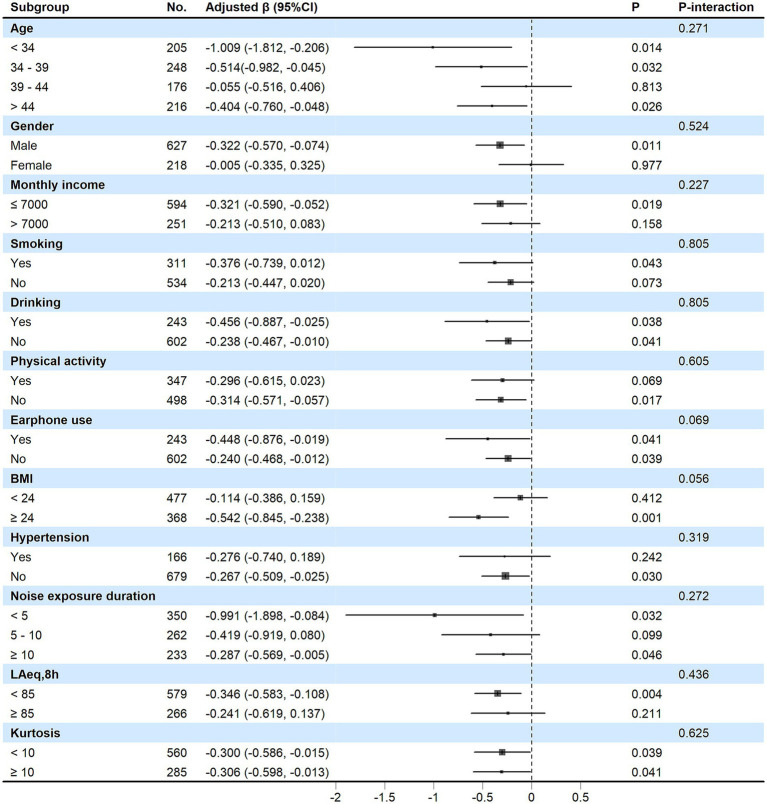
Subgroup analysis of the association between the HPD use duration and HF-PTA. Each group was adjusted for age, gender, monthly income, smoking, drinking, physical activity, earphone use, BMI, hypertension, exposure duration, L_Aeq,8h_, and kurtosis, except for the stratification variable.

As shown in Figure 3, no significant interactions were found across any of the subgroups (all *P*-interaction > 0.05), and consistent negative associations between HPD use duration and HF-PTA were observed.

### 3.5 Joint associations of noise exposure variables and HPD use duration with HF-PTA

**Figure 4:**
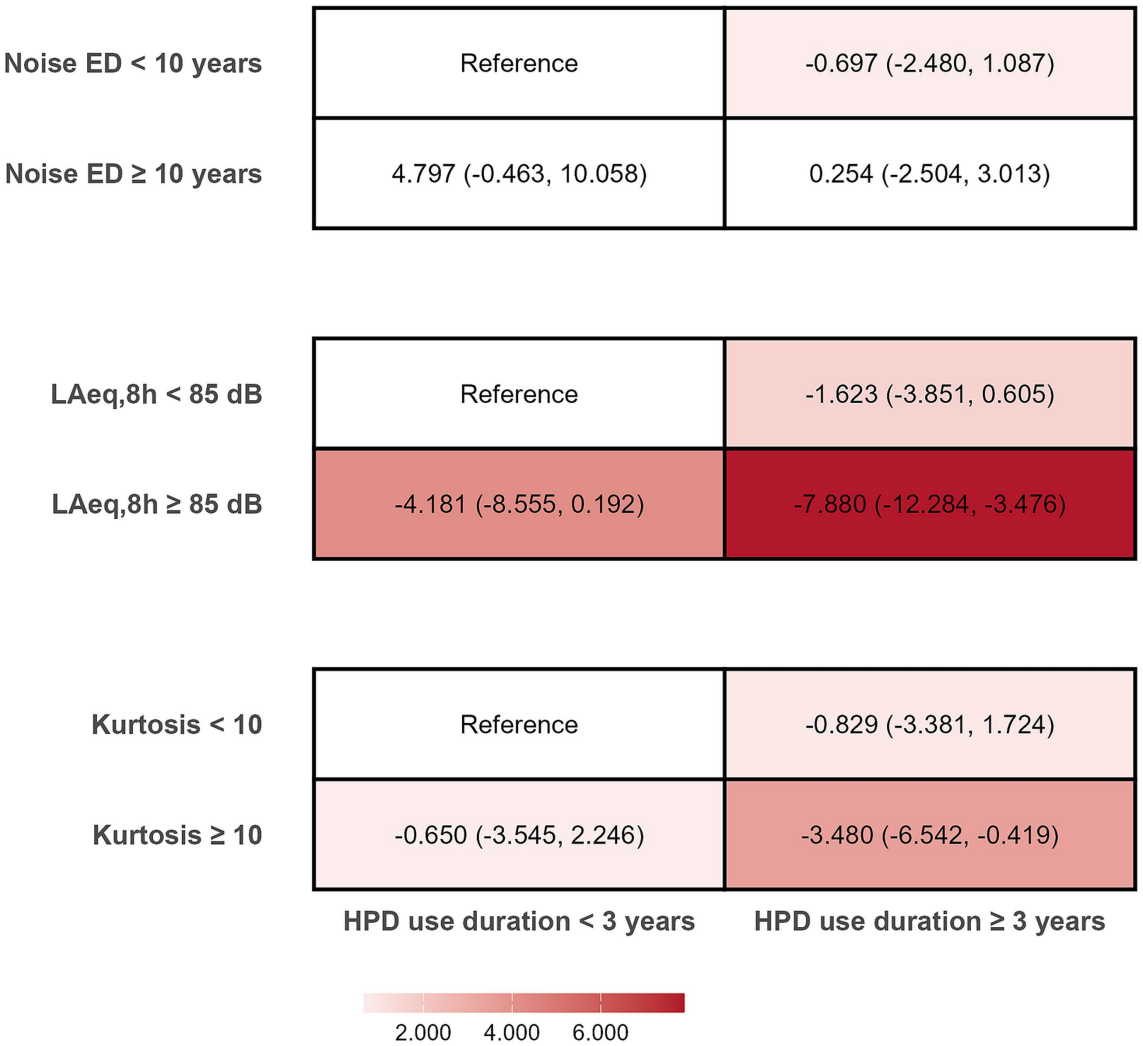
Joint associations of noise exposure duration, L_Aeq,8h_, kurtosis and HPD use duration with the risk of HF-PTA. Each group was adjusted for age, gender, monthly income, smoking, drinking, physical activity, earphone use, BMI, hypertension, exposure duration, L_Aeq,8h_, and kurtosis, except for the stratification variable. Values are β coefficient with 95% CI in parentheses. Noise ED, Noise exposure duration.

In Figure 4, compared with the respective reference groups, participants with L_Aeq,8h_ ≥ 85 dB and HPD use duration ≥ 3 years, as well as those with kurtosis ≥ 10 and HPD use duration ≥ 3 years, were linked to lower HF-PTA (*β* = −7.88, 95% CI: −12.28 to −3.48, *p* < 0.05; *β* = −3.48, 95% CI: −6.54 to −0.42, *p* < 0.05, respectively).

### 3.6 Discomfort causes

This study analyzed participants who reported discomfort with HPD use. Among HPD users (n = 763), 257 (33.68%) reported discomfort while using HPDs at work. The three most common reasons for discomfort were ear itching (114/257, 44.36%), ear fullness (79/257, 30.74%), and earache (58/257, 22.57%). Specific results are provided in Supplementary Table 2.

## 4 Discussion

To our knowledge, this study is the first to quantitatively assess the relationship between HPD use duration and NIHL risk while further exploring HPD-related factors associated with workers’ NIHL. In this cross-sectional study of manufacturing workers in Shenzhen, 845 adults aged 20–65 years from 24 factories were enrolled, combining an occupational health surveillance dataset with data collected from field-based questionnaires and noise exposure assessments. The results indicated that both enterprise-level factors (HPD provision and training) and individual-level factors (actual use, daily wearing frequency, HPD type, and cumulative use duration) were significantly associated with NIHL. Workers who had used HPDs for approximately 3 years or longer were associated with lower HF-PTA. However, 33.68% of workers reported discomfort while using HPDs, with ear itching being the most commonly reported cause.

Our finding highlights a relationship between enterprise-level interventions and lower HF-PTA, underscoring the potential role of organizational actions in occupational hearing conservation. Even when workers are aware of noise hazards, the provision of HPDs and regular training at the enterprise level may promote better compliance and proper usage, potentially enhancing the hearing protection. At the individual level, our findings further suggest that continuous HPD use throughout the workday was associated with lower HF-PTA. Similar to our results, a study by Saffree Jeffree et al. (43) indicated that infrequent HPD use was not associated with significant hearing protection. One possible explanation is that intermittent HPD use, even during substantial portions of the workday, may be associated with periods of unprotected exposure, which could be related to less favorable hearing outcomes. However, the inability to wear HPDs continuously during the workday may be due to the need for workers to communicate and interact during their tasks (44). Additionally, many workers reported that HPD use caused discomfort and a sense of isolation (45, 46), which may be linked to lower willingness to consistently wear HPDs throughout the workday. Moreover, earplug use, but not earmuff use, was significantly associated with a lower HF-PTA. This result may be due to the relatively small number of earmuff users in our study, leading to no statistically significant association observed for earmuff use; larger studies with better control for occupational differences are needed to verify its association. And this finding contradicts a previous systematic review, which concluded that both earplugs and earmuffs are effective in reducing NIHL risk (47). This finding may also be subject to some limitations, given the difficulty in obtaining detailed information on the specifications of earplugs and earmuffs used by individual participants.

For HPD use duration, this study quantitatively examined the dose–response relationship between cumulative HPD use duration and NIHL risk. Extending prior evidence that HPD use significantly reduces NIHL in occupational settings (48), a potential inflection point around 3 years was observed in the piecewise analysis, confirming the sustained protective value of long-term HPD use among workers. This aligns with previous findings suggesting that the association between HPD use and NIHL may be cumulative, with long-term, continuous use being associated with lower risk levels (29). A longitudinal study suggested that noise exposure and NIHL accumulate gradually (49), thus prolonged use of HPDs (approximately 3 years in the present study) may slow the progression of hearing loss. However, this finding should be interpreted cautiously. Our study also showed that in workplaces with high-intensity or high-impact noise, sustained HPD use for three or more years was linked to lower HF-PTA among workers. According to the Chinese national standard (GBZ 2.2–2007), the occupational exposure limit for harmful noise (L_Aeq,8h_) is 85 dB(A), above which noise is considered hazardous and requires both engineering controls and hearing protection measures. Previous evidence has indicated that higher kurtosis levels are significantly associated with more severe NIHL (10). Therefore, it is essential to identify high-risk workers timely and facilitate early intervention. From an occupational health perspective, policies emphasizing consistent and long-term HPD use may lead to lower NIHL risk among workers.

Lastly, given that HPDs are typically worn for extended periods during work, comfort is crucial to worker compliance and long-term willingness to use them (2). According to prior studies (50), the discomfort reported by workers in our survey primarily involved two aspects: physical comfort (ear fullness, ear itching, head fullness, earache) and functional comfort (slippage). Our finding that ear itching was the most common complaint aligns with a review on HPD comfort, with poor fit or slippage also being a significant concern in terms of functional comfort (51). Additionally, earaches are caused by the static mechanical pressure exerted by earplugs on the ear canal walls (51). To promote long-term, consistent use of HPDs, employers should prioritize comfort when selecting HPDs, and manufacturers should focus on ergonomic design.

This study addressed the association between HPD-related factors and NIHL, filling a gap in previous research by providing a quantitative assessment of HPD use duration. The findings suggest an approximately three-year point in HPD use, with longer and more consistent use being associated with lower HF-PTA. However, several limitations exist in this study. Firstly, in this study, the outcome examination preceded exposure assessment, with hearing thresholds obtained in 2024 and exposure information (questionnaires and noise recordings) collected in 2025. The analysis therefore relies on the assumption that characteristics represent prior exposure conditions, and the cross-sectional design restricts the ability to infer causality. And hearing thresholds in the WHED had already been age- and sex-corrected according to the national standard (GB/T 7582), and the original uncorrected audiometric data were not available. Accordingly, the outcome measure in this study reflects age- and sex-adjusted HF-PTA. Under this constraint, potential residual confounding related to correlations between age and noise exposure duration cannot be entirely excluded. Additionally, HPD use duration and related factors were self-reported by the workers, which introduces the potential for subjective bias and may influence the stability of the association results. This design, combined with self-reported data, could create a risk of reverse causation—for example, workers with existing hearing loss may be more likely to adopt or report consistent HPD use or change their behaviors after becoming aware of hearing loss, limiting causal inference regarding an “effective” usage duration. Thus, the approximately three-year inflection point identified in this study is subject to the aforementioned limitations, resulting in some uncertainty, and should be considered a preliminary and exploratory finding. To establish more robust associations, future studies should incorporate objective data and long-term follow-up, further evaluating HPD effectiveness through fit tests. Secondly, this study did not comprehensively assess non-occupational noise exposure. The absence of such data may introduce residual confounding, and future research should incorporate more detailed assessment of recreational exposure. Thirdly, this study focused on only six HPD-related factors. Future research could consider additional HPD-related factors (e.g., replacement frequency, proper-use assessments) and occupational hazards exposure (e.g., heat, vibration), to provide a more comprehensive understanding of their effects on NIHL. Lastly, the study was limited to one city, which may introduce regional and population biases. Therefore, larger studies covering broader regions and diverse populations are necessary to further validate these results.

## 5 Conclusion

This study highlights the value of incorporating quantitative assessments of HPD use duration into NIHL prevention strategies. The findings suggest that long-term HPD use is associated with lower HF-PTA, particularly among workers with three or more years of continuous use in China. Longitudinal studies are needed to confirm the timing and magnitude of benefit. Both enterprise-level measures and individual HPD use factors are likely to be key protective factors. These findings offer practical insights into HPD application, contributing to improved occupational hearing protection and better safeguarding of the auditory health of noise-exposed workers.

## Data Availability

The raw data supporting the conclusions of this article will be made available by the authors, without undue reservation.
