# Supplementary material for: Association between hearing protection device use and noise-induced hearing loss among manufacturing workers in China: a cross-sectional study
Source: Front Public Health. 2026 Apr 13;14:1787668. doi: 10.3389/fpubh.2026.1787668 (PMC13111276; doi:10.3389/fpubh.2026.1787668)
Supplement: Supplementary file 1 [file Supplementary_file_1.docx]

**Supplementary Methods 1.** Calculation of L_Aeq,T_ and Kurtosis

Previous studies have highlighted the importance of both energy (Leq) and kurtosis in evaluating the hearing hazards associated with complex noise exposure **[1-3]**.

In accordance with the Chinese standard (GBZ/T 189.8-2007), the noise energy indicator, L_Aeq,T_, in this study was calculated as follows:

$$L_{\mathrm{Aeq},T}=10\lg(\frac{1}{T}\sum_{i=1}^{n} T_{i}{10}^{0.1L_{\mathrm{Aeq},T_{i}}})\mathrm{dB}(A)$$

Where $L_{Aeq,T}$ is the equivalent continuous sound level over the entire day; $L_{Aeq,Ti}$ is the equivalent continuous sound level during the time period $T_{i}$; $T$ is the total duration of all time periods;
$T_{i}$ is the duration of the $i^{\mathrm{th}}$ time period; and $n$ is the total number of time periods.

Kurtosis (β) is a statistical measure that quantifies how the tails of a distribution differ from those of a Gaussian distribution. It is defined as the ratio of the fourth-order central moment to the squared second-order central moment of a distribution [1]. Kurtosis characterizes the temporal distribution of acoustic energy and is regarded as a key supplementary parameter in assessing NIHL caused by non-Gaussian noise. In this study, β was computed over non-overlapping 60-second time windows from the noise recordings to optimize both computational efficiency and accuracy in evaluating high-frequency NIHL. The geometric mean of the measured kurtosis values was then calculated and used as the kurtosis metric, as recommended by Tian et al. [4]. β can be calculated using the following formula:

$$\beta=\frac{m_{4}}{m_{2}}=\frac{\frac{1}{n}\sum_{i=1}^{n} \left( x_{i} - \bar{x})^{4} \right.}{\left( \frac{1}{n}\Sigma_{i=1}^{n}\left( x_{i} - \bar{x})^{2} \right. \right)^{2}}$$

Where β represents the biased kurtosis of the sound pressure at each 60-second interval, n is the number of samples in the 60-second time window, i is the i-th sample of the sound pressure, and x̅ is the sample mean.

$$\beta_{GEO}=(\prod_{i=1}^{N} \beta_{i})^{1/N}$$

Where β_GEO_ is the geometric average of the βᵢ values calculated for a sample of N values; N is the number of kurtosis values of βᵢ obtained from the full-shift noise exposure recording.

| **Supplementary Table 1. Descriptive statistical information on the characteristics of the 845 subjects in the statistical comparison cohort in each factory (n, number of subjects in each group; ±, plus/minus 1 standard deviation; -, minimum to maximum** | | | | | | |  |
| --- | --- | --- | --- | --- | --- | --- | --- |
|  |  |  |  |  |  |  |  |
| Factory | Region (district) | Industry | n | Age (yrs) | L_Aeq,8h_ (dBA) | HPD use duration (yrs) |  |
| Factory 01 | Pingshan | Machinery Manufacturing | 45 | 31-56 | 76.38-97.46 | 0-24 |  |
|  |  |  |  | 44.27 ± 7.87 | 87.39 ± 4.36 | 4.98 ± 5.11 |  |
| Factory 02 | Pingshan | Machinery Manufacturing | 26 | 27-56 | 81.2-95.31 | 0-20 |  |
|  |  |  |  | 46.35 ± 7.47 | 87.65 ± 3.63 | 5.04 ± 6.46 |  |
| Factory 03 | Pingshan | Smelting and Rolling | 38 | 20-57 | 78.59-100.26 | 1-29 |  |
|  |  |  |  | 41.24 ± 7.71 | 86.53 ± 5.72 | 9.32 ± 7.83 |  |
| Factory 04 | Pingshan | Pharmaceutical and Chemical | 16 | 34-55 | 82.06-102.61 | 0-19 |  |
|  |  |  |  | 46.19 ± 6.69 | 88.11 ± 5.23 | 8 ± 4.63 |  |
| Factory 05 | Pingshan | Metal Structure Manufacturing | 26 | 21.13-54.75 | 78.89-96.31 | 0-5 |  |
|  |  |  |  | 41.13 ± 8.36 | 86.61 ± 4.97 | 1.23 ± 1.11 |  |
| Factory 06 | Longhua | Non-metallic Mineral Products Manufacturing | 73 | 23.83-55.01 | 70.67-88 | 0-29 |  |
|  |  |  |  | 43.24 ± 6.88 | 77.51 ± 4.41 | 6.04 ± 5.36 |  |
| Factory 07 | Longgang | Machinery Manufacturing | 22 | 22-61 | 70.11-93.39 | 0-14 |  |
|  |  |  |  | 40.05 ± 9.65 | 81.62 ± 5.82 | 2.61 ± 3.44 |  |
| Factory 08 | Longgang | Pharmaceutical and Chemical | 18 | 27-54 | 80.12-95.81 | 1-10 |  |
|  |  |  |  | 43.39 ± 7.14 | 85.9 ± 4.17 | 3.72 ± 2.49 |  |
| Factory 09 | Longgang | Machinery Manufacturing | 17 | 21-55 | 82.85-94.49 | 1-13 |  |
|  |  |  |  | 39.18 ± 10.76 | 88.48 ± 4 | 3.15 ± 2.76 |  |
| Factory 10 | Longgang | Electrical, Communication and Instrument Manufacturing | 57 | 23-56 | 70.65-105.4 | 0-16 |  |
|  |  |  |  | 38.46 ± 7.2 | 85.18 ± 5.83 | 4.24 ± 3.2 |  |
| Factory 11 | Longgang | Machinery Manufacturing | 10 | 21-37 | 80.57-87.06 | 0-8 |  |
|  |  |  |  | 27.9 ± 6.81 | 83.65 ± 2.25 | 1 ± 2.54 |  |
| Factory 12 | Guangming | Pharmaceutical and Chemical | 2 | 29-41 | 78.83-84.13 | 3-5 |  |
|  |  |  |  | 35 ± 8.49 | 81.5 ± 3.75 | 4 ± 1.41 |  |
| Factory 13 | Guangming | Electrical, Communication and Instrument Manufacturing | 25 | 24-44 | 72.93-88.67 | 1-11 |  |
|  |  |  |  | 35.64 ± 5.12 | 84.24 ± 3.26 | 4.18 ± 2.81 |  |
| Factory 14 | Guangming | Machinery Manufacturing | 8 | 30-50 | 82.38-89.41 | 1-6.5 |  |
|  |  |  |  | 42.38 ± 6.7 | 84.75 ± 2.61 | 3.25 ± 2.1 |  |
| Factory 15 | Guangming | Electrical, Communication and Instrument Manufacturing | 86 | 23-58 | 70-92.09 | 0-20 |  |
|  |  |  |  | 39.83 ± 7.8 | 81.36 ± 5.07 | 7.49 ± 5.21 |  |
| Factory 16 | Guangming | Machinery Manufacturing | 13 | 22-50 | 73.95-86.12 | 0-9 |  |
|  |  |  |  | 36.92 ± 8.02 | 81.22 ± 3.45 | 2.65 ± 2.72 |  |
| Factory 17 | Guangming | Machinery Manufacturing | 39 | 23-56 | 79.01-97.42 | 1-14 |  |
|  |  |  |  | 43.18 ± 7.53 | 88.66 ± 4.28 | 5.65 ± 3.54 |  |
| Factory 18 | Guangming | Electrical, Communication and Instrument Manufacturing | 61 | 21-49 | 70.1-84.59 | 0-13 |  |
|  |  |  |  | 35.2 ± 6.57 | 76.56 ± 3.36 | 4.23 ± 2.64 |  |
| Factory 19 | Guangming | Electrical, Communication and Instrument Manufacturing | 42 | 23-44 | 70.47-83.43 | 0-16 |  |
|  |  |  |  | 35.12 ± 4.76 | 75.41 ± 3.41 | 1.58 ± 3.59 |  |
| Factory 20 | Guangming | Smelting and Rolling | 18 | 32-54 | 79.84-90.64 | 1-16 |  |
|  |  |  |  | 41.72 ± 5.95 | 82.79 ± 2.53 | 7.5 ± 4.47 |  |
| Factory 21 | Guangming | Electrical, Communication and Instrument Manufacturing | 29 | 25-50 | 72.32-90.21 | 0-15 |  |
|  |  |  |  | 40.21 ± 6.42 | 83.44 ± 3.94 | 5.74 ± 4.71 |  |
| Factory 22 | Guangming | Machinery Manufacturing | 75 | 20-44 | 70.18-91.22 | 0-21 |  |
|  |  |  |  | 34.15 ± 5.38 | 79.75 ± 4.87 | 6.39 ± 5.18 |  |
| Factory 23 | Guangming | Electrical, Communication and Instrument Manufacturing | 15 | 28-40 | 72.03-96.92 | 0-8 |  |
|  |  |  |  | 34.53 ± 4.03 | 82.23 ± 9.08 | 3.63 ± 2.43 |  |
| Factory 24 | Guangming | Metal Structure Manufacturing | 84 | 23.7-49.27 | 75.54-87.4 | 0-9 |  |
|  |  |  |  | 35.72 ± 5.5 | 81.48 ± 2.27 | 2.27 ± 1.77 |  |


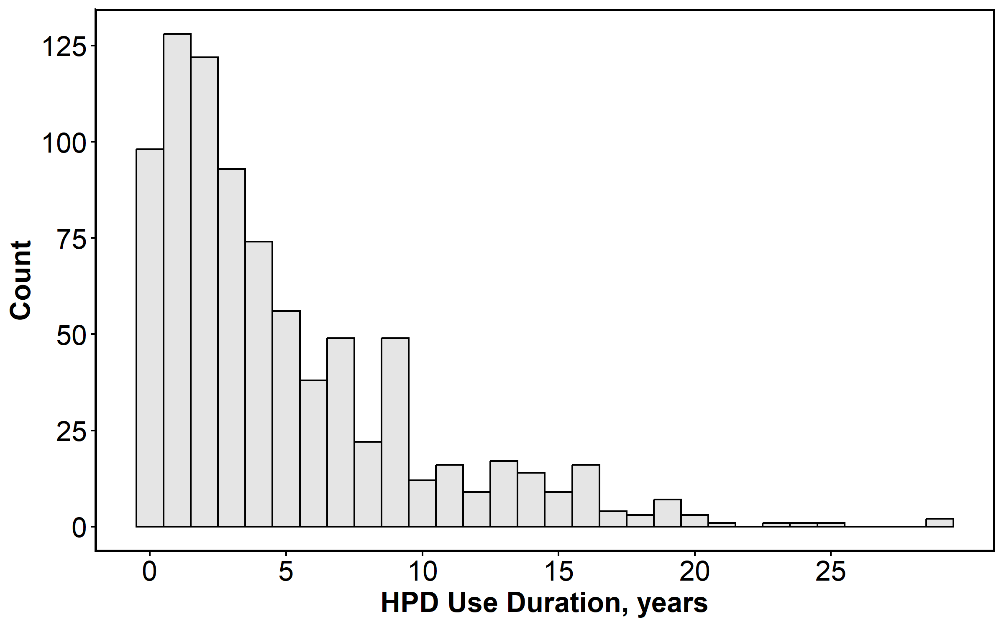


**Supplementary Figure 1.** Count distribution of HPD use duration

**Supplementary Table 2.** The comfort situation and proportion of discomfort causes

| **HPD comfort related variables** | **n** | **%** |
| --- | --- | --- |
| **Comfort Status** |  |  |
| Yes | 506 | 66.32 |
| No | 257 | 33.68 |
| **Discomfort Reasons (multiple responses)** |  |  |
| Ear fullness | 79 | 30.74 |
| Ear itching | 114 | 44.36 |
| Head fullness | 22 | 8.56 |
| Earache | 58 | 22.57 |
| Slippage | 43 | 16.73 |

***References:***

1. Zhang, MB, Qiu, W, Xie, HW, Xu, X, Shi, Z, Gao, X, et al. Applying Kurtosis as an Indirect Metric of Noise Temporal Structure in the Assessment of Hearing Loss Associated With Occupational Complex Noise Exposure. Ear Hear. (2021) 42:1782-1796. doi:10.1097/aud.0000000000001068

2. Qiu, W, Murphy, W, Suter, A. Kurtosis: A New Tool for Noise Analysis. Acoustics Today. (2020) 16:39-47. doi:10.1121/AT.2020.16.4.39

3. Zhang, MB, Xie, HW, Zhou, JN, Sun, X, Hu, W, Zou, H, et al. New Metrics Needed in the Evaluation of Hearing Hazard Associated With Industrial Noise Exposure. Ear Hear. (2021) 42:290-300. doi:10.1097/aud.0000000000000942

4. Tian, Y, Ding, WX, Zhang, MB, Zhou, T, Li, J, Qiu, W. Analysis of correlation between window duration for kurtosis computation and accuracy of noise-induced hearing loss prediction. J Acoust Soc Am. (2021) 149:2367. doi:10.1121/10.0003954
